# Development of Microfluidic, Serum-Free Bronchial Epithelial Cells-on-a-Chip to Facilitate a More Realistic *In vitro* Testing of Nanoplastics

**DOI:** 10.3389/ftox.2021.735331

**Published:** 2021-10-06

**Authors:** Govind Gupta, Srikanth Vallabani, Romain Bordes, Kunal Bhattacharya, Bengt Fadeel

**Affiliations:** ^1^ Unit of Molecular Toxicology, Institute of Environmental Medicine, Karolinska Institutet, Stockholm, Sweden; ^2^ Unit of Biochemical Toxicology, Institute of Environmental Medicine, Karolinska Institutet, Stockholm, Sweden; ^3^ Department of Chemistry and Chemical Engineering, Chalmers University of Technology, Göteborg, Sweden

**Keywords:** alternative methods, *in vitro*, microfluidics, nanoplastics, nanotoxicology

## Abstract

Most cell culture models are static, but the cellular microenvironment in the body is dynamic. Here, we established a microfluidic-based *in vitro* model of human bronchial epithelial cells in which cells are stationary, but nutrient supply is dynamic, and we used this system to evaluate cellular uptake of nanoparticles. The cells were maintained in fetal calf serum-free and bovine pituitary extract-free cell culture medium. BEAS-2B, an immortalized, non-tumorigenic human cell line, was used as a model and the cells were grown in a chip within a microfluidic device and were briefly infused with amorphous silica (SiO_2_) nanoparticles or polystyrene (PS) nanoparticles of similar primary sizes but with different densities. For comparison, tests were also performed using static, multi-well cultures. Cellular uptake of the fluorescently labeled particles was investigated by flow cytometry and confocal microscopy. Exposure under dynamic culture conditions resulted in higher cellular uptake of the PS nanoparticles when compared to static conditions, while uptake of SiO_2_ nanoparticles was similar in both settings. The present study has shown that it is feasible to grow human lung cells under completely animal-free conditions using a microfluidic-based device, and we have also found that cellular uptake of PS nanoparticles aka nanoplastics is highly dependent on culture conditions. Hence, traditional cell cultures may not accurately reflect the uptake of low-density particles, potentially leading to an underestimation of their cellular impact.

## Introduction

Experts in the field have argued that “nanotoxicology is currently at a crossroads and faces a number of obstacles and technical limitations not associated with traditional toxicology” (Hussain et al., 2015). In fact, the field of nanotoxicology still relies heavily on assays and methods developed for the testing of traditional chemicals, and the development of relevant and robust assays amenable to high-throughput screening of nanomaterials represents an important priority (Li et al., 2018; Fadeel, 2019). There is a strong consensus that faster and animal-free approaches for safety assessment of chemicals as well as engineered nanomaterials are needed (Kohl et al., 2021). Conventional cell culture models fail to recapitulate the dynamic environment of a living system, and microfluidic cell culture systems have emerged in recent years as a promising alternative with the potential to replace or at least reduce the use of animal experiments (Bhatia and Ingber, 2014; Ingber, 2020). Huh et al. (2010) developed a mechanically active lung-on-a-chip device and were able to demonstrate that cyclic mechanical strain to simulate breathing accentuates the toxicity of silica nanoparticles (NPs). More recent developments include the design of multiorgan-on-a-chip devices in an attempt to capture the crosstalk between different cell types (Ashammakhi et al., 2020). Additionally, recent attempts have been made to grow tumor spheroids in a microfluidic device to more accurately model and determine NP uptake (Zhuang et al., 2019).

Using microfluidics-based cell culture systems, several investigators have provided evidence that NPs may display different effects under dynamic flow conditions as opposed to conventional, static cell culture conditions. For instance, Kim et al. (2011) investigated the cytotoxicity of mesoporous silica NPs towards immortalized human endothelial cells under flow conditions and found that the NPs showed higher toxicity under flow conditions when compared to static conditions. In contrast, Fede et al. (2015) evaluated the toxicity of ultrasmall gold NPs towards human umbilical vein endothelial cells (HUVEC) under static and flow conditions and found that the toxicity was reduced under flow conditions. In the latter study, uptake of NPs under flow was found to be lower than in static conditions. Using a panel of cancer cell lines, Kang et al. (2016) showed that cellular uptake of polystyrene NPs (100 nm) was higher under shear stress conditions when compared to static cell cultures. Other investigators have shown, using a panel of solid vs. hollow silica NPs of roughly the same size (350 nm), that the particle density affected cellular uptake and toxicity under flow conditions (Yazdimamaghani et al., 2018). Xu et al. (2020) developed a lung-on-a-chip model consisting of endothelial cells and epithelial cells to recapitulate the alveolar-blood barrier (to study fine particulate matter). However, the divergent outcomes of these studies suggest that important differences exist not only between static and dynamic cell culture conditions, but also depending on the cell types used and on the types of NPs.

Plastics have outpaced most man-made materials yet none of the commonly used plastics are biodegradable, and plastic debris therefore accumulates in the environment (Geyer et al., 2017). Consequently, numerous studies have addressed the environmental impact of plastic litter and microplastic fragments (for a review, see Mitrano et al., 2021). However, few studies have focused on the potential human health effects of microplastics or the nanoscale breakdown products commonly referred to as nanoplastics. Most *in vitro* toxicological studies use polystyrene (PS) NPs as model particles and, for the most part, toxicity is only observed at high concentrations or following long-term exposure, unless the NPs are endowed with a positive surface charge, as is the case for amino-functionalized PS NPs which were shown to be toxic towards a range of cell types (Anguissola et al., 2014; Mrakovcic et al., 2014; Ruenraroengsak and Tetley, 2015; Hesler et al., 2019; He et al., 2020). Notwithstanding the fact that spherical NPs may not be representative of the heterogenous features of plastic debris (Gigault et al., 2021), the question remains whether traditional cell culture models are suitable for the evaluation of nanoplastics.

Cho et al. (2011) investigated cellular uptake of gold NPs using upright and inverted cell culture configurations and found that uptake depends on the sedimentation and diffusion velocities and is independent of size, shape, density, and surface coating of the NPs. Thus, the toxicologically relevant dose should take sedimentation into account (Lison and Huaux, 2011). However, the gold NPs used in the latter study may not accurately reflect the behavior of low-density nanoplastics. In fact, Watson et al. (2016) showed in a seminal study that the toxicity of low-density NPs may be overlooked when using conventional cell culture models. The authors tested polypropylene (PP) NPs and compared conventional and inverted cell culture platforms using primary human monocyte-derived macrophages maintained in standard medium supplemented with fetal bovine serum (FBS). No toxicity was observed in the conventional set-up whereas a dose-dependent decrease in cell viability and an increase in reactive oxygen species production was observed in the inverted cell culture system. The authors argued that due to the buoyancy of the NPs, there is “essentially zero dose delivered to the cells at the bottom of the well” when administering the particles in the conventional model (Watson et al., 2016). However, not all cells can be maintained upside-down in culture, and a model in which a dynamic flow is applied using a microfluidics-based system may be a more relevant way of addressing low-density NPs such as nanoplastics. The present study seeks to develop a more realistic *in vitro* model of the human lungs based on serum-free culture of a human lung cell line using cell culture-on-a-chip microfluidic technology. We prepared a step-by-step guide for the assembly of the test platform, which can be found in the supplement accompanying this paper. Furthermore, as a proof-of-concept, we studied fluorescent silica NPs and PS NPs of similar size (45 and 50 nm) and determined cellular uptake under static vs. dynamic cell culture conditions using the BEAS-2B cell line.

## Materials and Methods

### Microfluidic System and Cell Culture-on-a-Chip Model

A step wise description of the assembly of the microfluidic system is provided in the Supplementary Box 1. The system is comprised of five different parts, including the cell culture-on-a-chip (COC) procured from Micronit (Netherlands). The resealable top and bottom layers are of same width (15 mm) and length (45 mm) as the 0.4 mm glass middle layer. Assembly of top and bottom layers resulted in the formation of two flow chambers separated by a middle layer that contains the cell culture membrane. Hence, a cavity with a polyester (PET) membrane fixed on a glass slide, with a thickness of 12 µm and pore size of 0.45 µm, with 1.6 × 10^6^ pores/cm^2^ density and 1.6 cm^2^ surface area, separates the upper chamber (UC) and lower chamber (LC). Both upper and lower slides were spaced from the middle glass layer membrane *via* a silicone gasket (0.25 mm), resulting in a volume of 110 and 75 µL for the UC and LC, respectively, and a total volume of 185 µL for the device. The created distance from the middle layer to either top or bottom layer was 0.25 mm whereas the distance between the top layer and the membrane on middle layer cavity was 0.65 mm. The chip was mounted with a quick locking mechanism in the chip holder constructed for connecting external tubing to the chip *via* ferrules to ensure tight connections and a leak-free system. The specified NPs were added to two separate input glass bottles (50 ml) connected through the digitally operated OB1 MK3+ pressure controller by Elveflow (Elvesys, France). The glass bottles were then connected to the UC and LC compartments of the COC with polytetrafluoroethylene (Teflon) tubing (0.25 mm inner diameter, 14.5 cm length) through the Fluidic Connect Pro chip holder (Micronit). The same tubing was used on the outlets of the chip to connect with the glass collecting reservoirs. Prior to cell culture experiments, all tubing and chip parts were placed under the UV light in a laminar air flow and sterilized using 70% ethanol and the tubing was filled with medium to eliminate air bubbles. The microfluidic system was placed in an incubator at 37°C to sustain cell culture conditions.

### Animal-Free Culture of Human Bronchial Epithelial Cells

The immortalized human bronchial epithelial cell line, BEAS-2B (European Collection of Cell Cultures) was cultured in PneumaCult™-Ex Plus Medium (Stemcell Technologies, United Kingdom) supplied with 50x extra supplement; hydrocortisone (Stemcell Technologies, United Kingdom) and penicillin-streptomycin solution (Gibco, Sweden) was added to the complete cell medium. It is important to note that the cell medium is free from FBS and bovine pituitary extract (BPE). Hence, the cell culture medium can be considered “animal-free” (Oredsson et al., 2019). Furthermore, BEAS-2B cells are often grown on a substrate of fibronectin, collagen, and serum albumin of bovine origin. However, we were able to maintain cells without pre-coating with extracellular matrix proteins (Supporting Information), thus avoiding the use of animal proteins. Hence, the cells were seeded in 75 cm^2^ tissue culture flasks without pre-coating and expanded until 70–80% confluence for further studies under static or dynamic conditions, as described below.

### Fluorescence Microscopy and Cell Viability Assessment

*Cell imaging*: For optical and fluorescence microscopy, cells were seeded overnight on glass coverslip placed in a 24-well plate or in the microfluidic chip. Next, cells washed with PBS and fixed with paraformaldehyde (4%). Thereafter, cells were washed and stained with CellMask™ Deep Red to visualize the plasma membrane (Thermo Fisher Scientific) and counterstained and mounted with ProLong™ Gold Antifade Mountant containing DAPI to visualize cell nuclei (Thermo Fisher Scientific) and imaged using the EVOS™ M7000 imaging system (Thermo Fisher Scientific) at 400x magnification. *Cell viability*: BEAS-2B cells were seeded at a density of 60,000 cells/cm^2^ either in a 24-well plate or on the microfluidic chip. Cell supernatants were collected 24 h after seeding and LDH release was measured for cell viability assessment by using the CytoTox96^®^ Non-Radioactive Cytotoxicity Assay kit (Promega).

### Preparation of Silica and Polystyrene Nanoparticles

FITC-labelled colloidal SiO_2_ NPs (primary size 45 nm, density ∼2.0 g/cm^3^) and Dragon Green™-labelled polystyrene (PS) nanoparticles (primary size 50 nm; density ∼1.06 g/cm^3^) were used in the present study. Fluorescent SiO_2_ NPs were prepared using a modified Stöber synthesis (Pihl et al., 2019). In brief, a fluorescent precursor was prepared by reacting fluorescein isothiocyanate (FITC) with (3-aminopropyl)trimethoxysilane. This conjugate was then condensed with tetraethyl orthosilicate (TEOS) to yield fluorescent particles in a mixture of water, ethanol and ammonia. The particles were purified and further coated with an extra layer of silica. An advantage of this approach is that the fluorophore is incorporated in the core of the NPs thus preventing leakage of the dye while preserving the surface chemistry of the NPs. The FITC-SiO_2_ NPs were used for experiments after dilution at the desired concentrations in cell culture medium. The PS NPs were obtained from Bangs Laboratories, Inc. (USA). According to the manufacturer, the latter NPs were produced by embedding the fluorescent dye internally leaving the surface groups of the NPs unaltered. However, the colloidal suspension (1% solid or 10 mg/ml) of PS NPs contained surfactant (<0.1%) as well as biocide (NaN_3_) (<0.09%). This may obviously skew the results (Heinlaan et al., 2020). Therefore, the NPs were dialyzed for 3 days using the 10 kDa gamma-irradiated Slide-A-Layer™ dialysis cassettes (ThermoScientific). Water was changed every day during dialysis for three consecutive days. The SiO_2_ NP and PS NP stock solutions were dispersed in cell culture medium at 10 μg/ml and gently vortexed for 30 s prior to the experiments with BEAS-2B cells. For characterization, NPs were collected from both inlet and outlet reservoirs connected to the microfluidics system. After 1 h of exposure, the samples were collected from inlet and outlet reservoirs and characterization was performed. Hydrodynamic diameter and ζ-potential measurements were performed as described previously (Bhattacharya et al., 2017) using the Zetasizer Nano ZS90 (Malvern, UK).

### Exposure of Cells Under Dynamic and Static Conditions

A step wise description of the assembly of the cell culture-on-a-chip (COC) is shown in the Supplementary Box 2. Briefly, BEAS-2B cells were seeded 1 day before the experiment at a density of 60,000 cells/cm^2^ either in a 24-well plate or on the microfluidic glass chip for static and dynamic exposures, respectively. The cells grown in 24-well plates and the microfluidic chip were monitored for cell morphology and for cell viability by measuring LDH release, as detailed above. Then, the microfluidic chip was inserted and sealed with top and bottom layers to assemble the COC system. Exposure to SiO_2_ and PS NPs (10 μg/ml) was performed under static and dynamic conditions (flow rate: 65 μL/min, shear stress: 0.015 dyne/cm^2^). This was achieved by applying a positive pressure (400 ± 7 mbar) in the microfluidic system. The cells were exposed both through the upper and lower channel of the system. For some experiments, exposure was performed separately through upper or lower channel, respectively. After exposure, the samples were collected by trypsinization (0.025%) and fixed with 4% paraformaldehyde for analysis of cellular uptake, see below.

### Uptake of Fluorescent Particles by Human Lung Cells

*Flow cytometry:* The cellular association of SiO_2_ NPs or PS NPs with BEAS-2B cells was quantified by measurement of FITC fluorescence by flow cytometry. In brief, the cells were washed thrice and resuspended on HBSS medium and fluorescence intensity was measured using BD LSRFortessa™ flow cytometer (BD Biosciences) operating with BD FACS DIVA™ software (BD Biosciences). The cell population was gated on the basis of side scatter (SSC) and forward scatter (FSC) intensities detected in control samples. To avoid interference from residual NPs or cellular debris, a FSC threshold was set with a cutoff value of 5,000. The data were plotted using FCS Express™ v. 7 Flow Cytometry software and presented in the form of histograms showing a change in fluorescence intensity after NP exposure compared to control. *Confocal microscopy:* To validate the cellular uptake of NPs, samples harvested as described above were analyzed by confocal microscopy. The use of fluorescent NPs and fluorescent dyes precluded the need for antibodies. The formaldehyde fixed cells were washed and stained with phalloidin red (Abcam) for 15 min and counterstained and mounted with ProLong™ Gold Antifade Mountant containing DAPI (Thermo Fisher Scientific) and imaged using a Zeiss LSM880 confocal microscope. Data were also collected along the *z*-axis, and images were further processed using ZEN software (Zeiss).

## Results

### Cell Culture-on-a-Chip for Assessment of Particle Uptake

**FIGURE 1:**
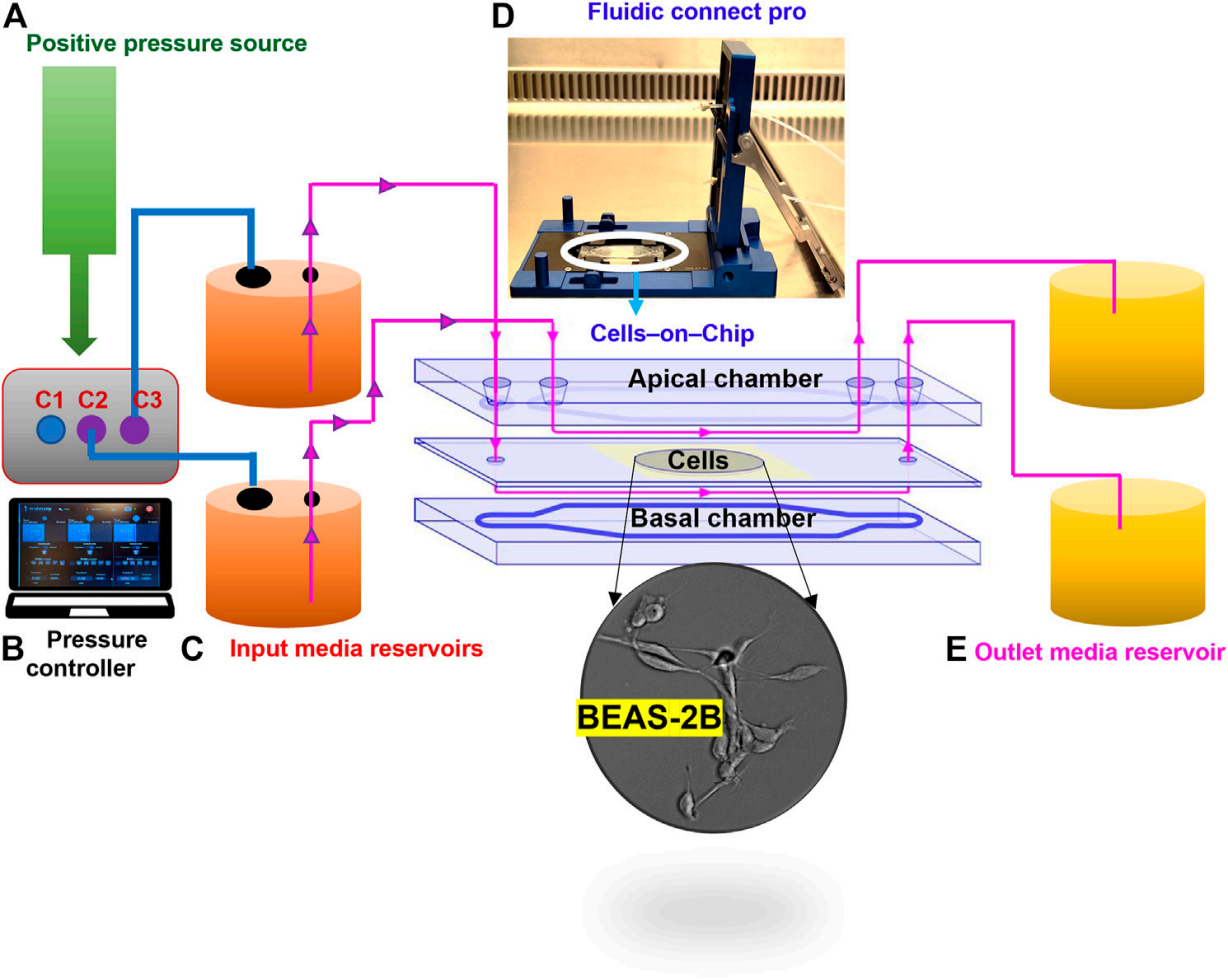
Configuration of the microfluidic cell culture-on-a-chip (COC) platform. The device consists of five different parts: clean air supplied as a positive pressure source **(A)**, for the digitally operated pressure controller with three defined channels to sustain maximum pressures; 10 bar in C1 and 1 bar in C2 and C3 **(B)**; then, the desired amount of pressure supplied to the inlet reservoirs through tubing **(C)**, which enables the flow of medium through the fluidics connect pro device that holds the COC chambers to allow unidirectional flow of medium through the apical and basal sides of the human lung cells in the central cavity **(D)**; finally, flow through is collected in outlet reservoir **(E)**.

We established a microfluidic-based, serum-free bronchial epithelial cells-on-a-chip system for the evaluation of cellular uptake of NPs of differing density. The step wise assembly of the system is described in the Supplementary Box 1,2. The configuration of the cell culture-on-a-chip (COC) device is depicted in Figure 1. For comparison, cells were maintained in a conventional, 24-well cell culture dish. To allow for the comparison between the two set-ups, we first determined the cell density, cell viability, and cell morphology of the BEAS-2B cells cultured in 24-well cell culture plates vs. in the microfluidic device (Supplementary Table S1, Figure S1). Notably, cells were seeded at the same cell density in both systems and a low and comparable loss of cell viability (∼5%) was noted under both conditions at 24 h. BEAS-2B is a virally transformed yet non-tumorigenic human cell line (Reddel et al., 1988). These cells have been widely used as an *in vitro* model for assaying chemicals and nanomaterials with respect to pulmonary toxicity or carcinogenicity. The BEAS-2B cells were maintained and exposed under completely animal-free conditions in this study meaning that no animal-derived products (such as fetal bovine serum) were applied.

It is well understood that wherever flow occurs (in the body), shear stress exists. Thus, the respiratory epithelium is continuously subjected to shear stresses induced by airflow. It has been estimated that the shear stress values in the nasal cavity during quiet breathing are in the range of 0.5–1.5 dyne/cm^2^ (Elad et al., 2006). Higher values may occur as breathing efforts are increased and these may approach the shear stress values that exist in large blood vessels (Elad et al., 2006). Here we applied a constant flow rate (65 μL/min) in the microfluidic system to achieve a shear stress of 0.015 dyne/cm^2^. This value is lower than the ones reported for human airways. However, it is noted that the BEAS-2B cells were grown without pre-coating of the substrate with extracellular matrix proteins, as reported previously by others (*e.g.*, Zhao and Klimecki, 2015).

### Characterization of Silica and Polystyrene Nanoparticles

**FIGURE 2:**
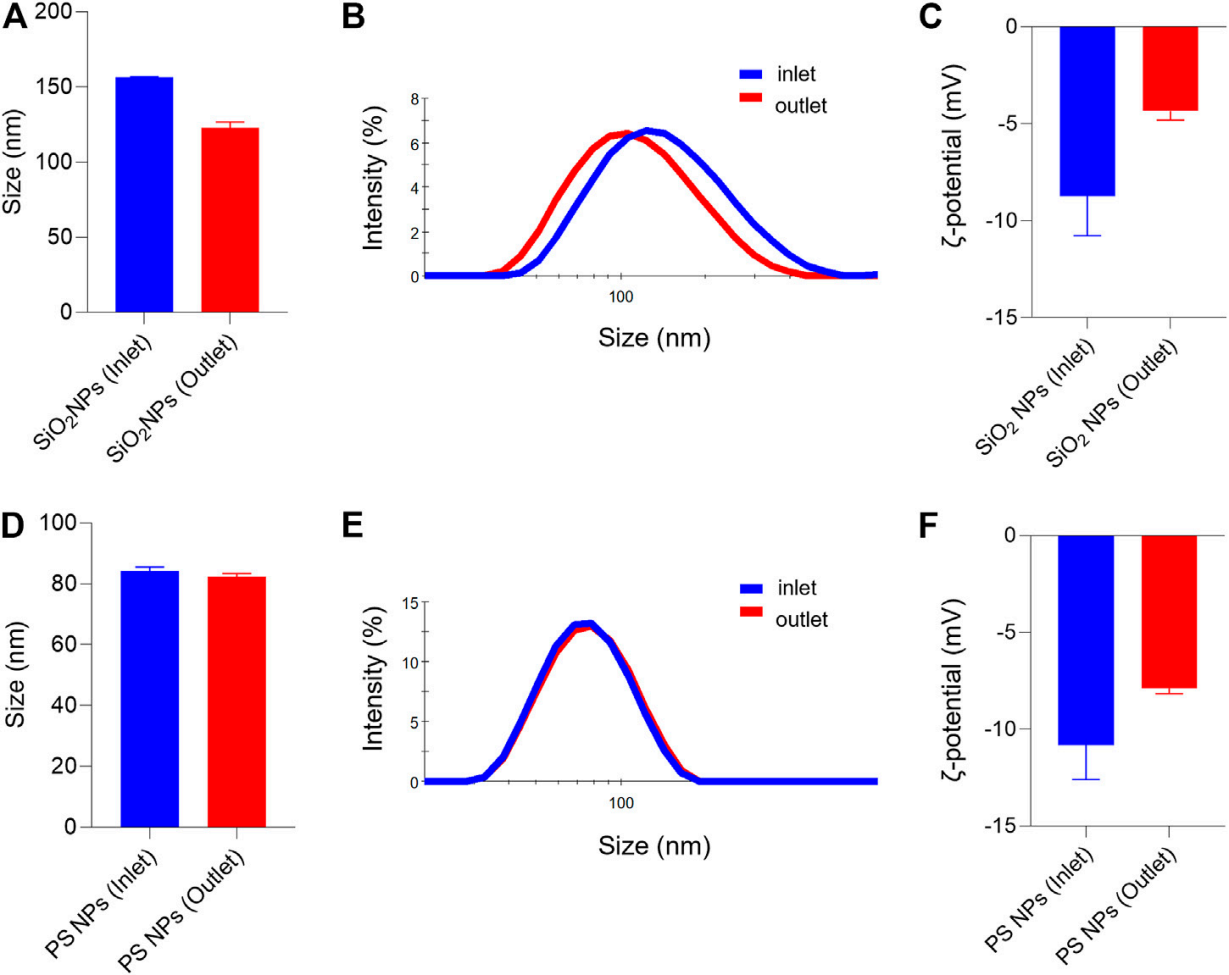
Hydrodynamic diameter and surface charge (ζ-potential) of SiO_2_ NPs and PS NPs collected from inlet and outlet reservoirs of the microfluidic device **(A–C)** SiO_2_ NPs were evaluated at 2 h of exposure **(D–F)** PS NPs were evaluated after 1 h of exposure. The hydrodynamic diameter of the SiO_2_ NPs was: D_h_ (inlet) = 156 ± 0.5, and D_h_ (outlet) = 123 ± 4; and for the PS NPs: D_h_ (inlet) = 84 ± 1.3; and D_h_ (outlet) = 82 ± 1.0.

Both SiO_2_ NPs and PS NPs are widely studied with respect to their biological behavior (Tenzer et al., 2013). Prior to the assessment of cellular uptake, we characterized the NPs in the relevant cell culture medium. Furthermore, we decided to evaluate the NPs before and after passing through the COC (refer to the schematic in Figure 1). It is noted that the cell culture medium in the present study is serum-free, yet it remains possible that proteins secreted by the cells, or other cellular metabolites, might influence the NPs (Albanese et al., 2014). Based on the information provided by the suppliers, the SiO_2_ NPs and PS NPs displayed similar primary particle sizes (45 and 50 nm, respectively). However, the NPs differed in terms of their density (SiO_2_ NPs ∼2.0 g/cm^3^; PS NPs ∼1.06 g/cm^3^). Dynamic light scattering (DLS) measurements showed that the hydrodynamic diameter of the SiO_2_ NPs in inlet and outlet samples (following 1 h exposure) was 156 ± 1 nm and 123 ± 4 nm, respectively (Figures 2A,B). In contrast, the hydrodynamic diameter of PS NPs remained almost identical in the outlet samples (82 ± 1 nm) as compared to the inlet samples (84 ± 1 nm) (Figures 2D,E). Furthermore, the ζ-potential was affected both in the case of the SiO_2_ NPs and PS NPs when collected from the outlet in comparison to the inlet reservoir, more so for the SiO_2_ NPs (Figures 2C,F). Hence, the ζ-potential of the SiO_2_ NPs was −8.7 ± 2.0 mV and −4.3 ± 0.5 mV in the inlet and outlet samples, respectively (Figure 2C) whereas for PS NPs, the ζ-potential was −10.8 ± 1.8 mV and −7.9 ± 0.3 mV in the inlet and outlet samples, respectively (Figure 2F).

### Dynamic Exposure Conditions Affect Uptake of Particles

**FIGURE 3:**
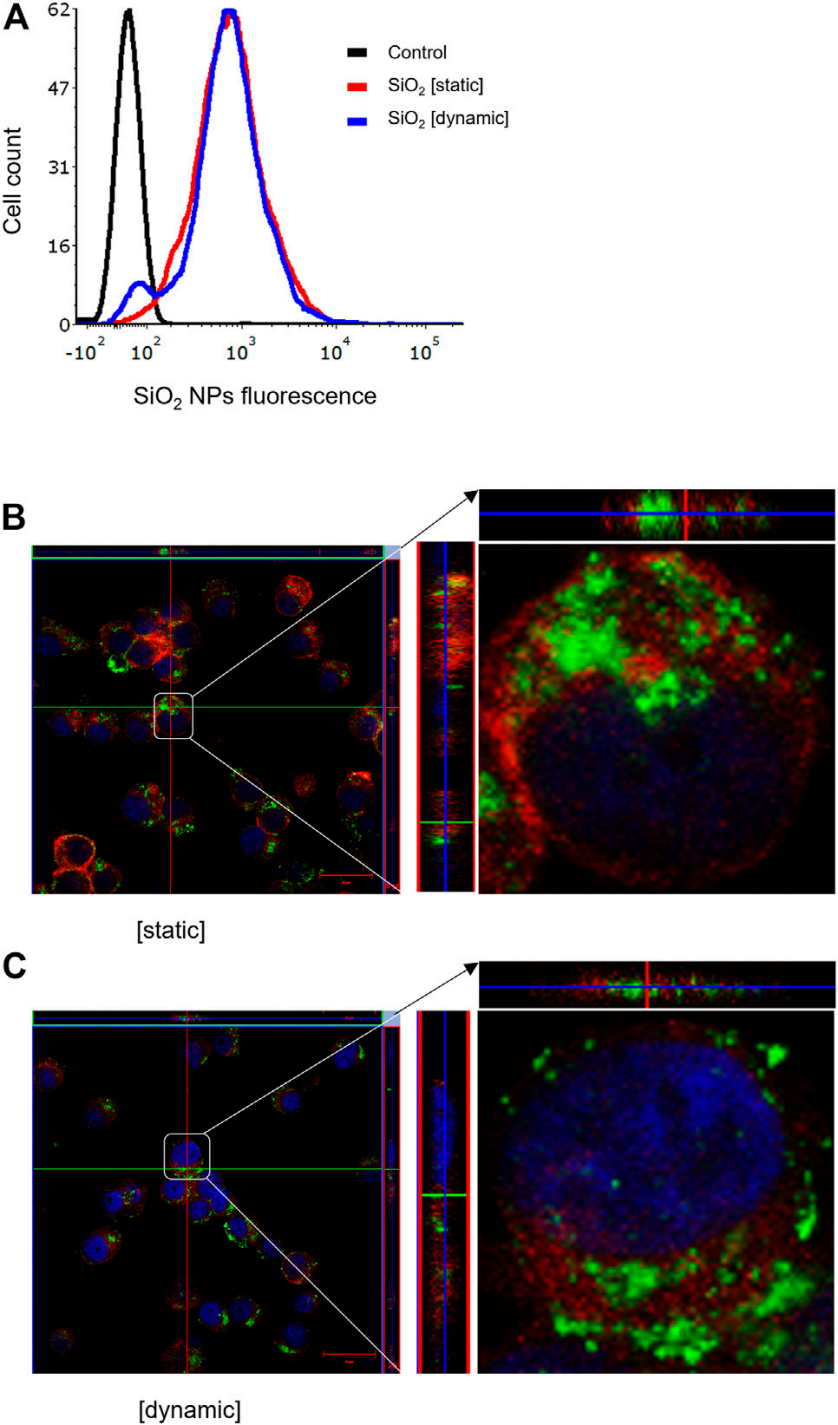
Cellular uptake of fluorescent SiO_2_ NPs (10 μg/ml) under static and dynamic exposure conditions. **(A)** Cellular uptake of SiO_2_ NPs determined using flow cytometry after 2 h of exposure of BEAS-2B cells **(B,C)** Confocal microscopy of cells exposed for SiO_2_ NPs after 2 h under static and dynamic exposures, respectively. The z-stack imaging confirmed the intracellular presence of the NPs. Cells are counterstained using phalloidin red and DAPI (blue) to visualize the actin cytoskeleton and cell nucleus.

**FIGURE 4:**
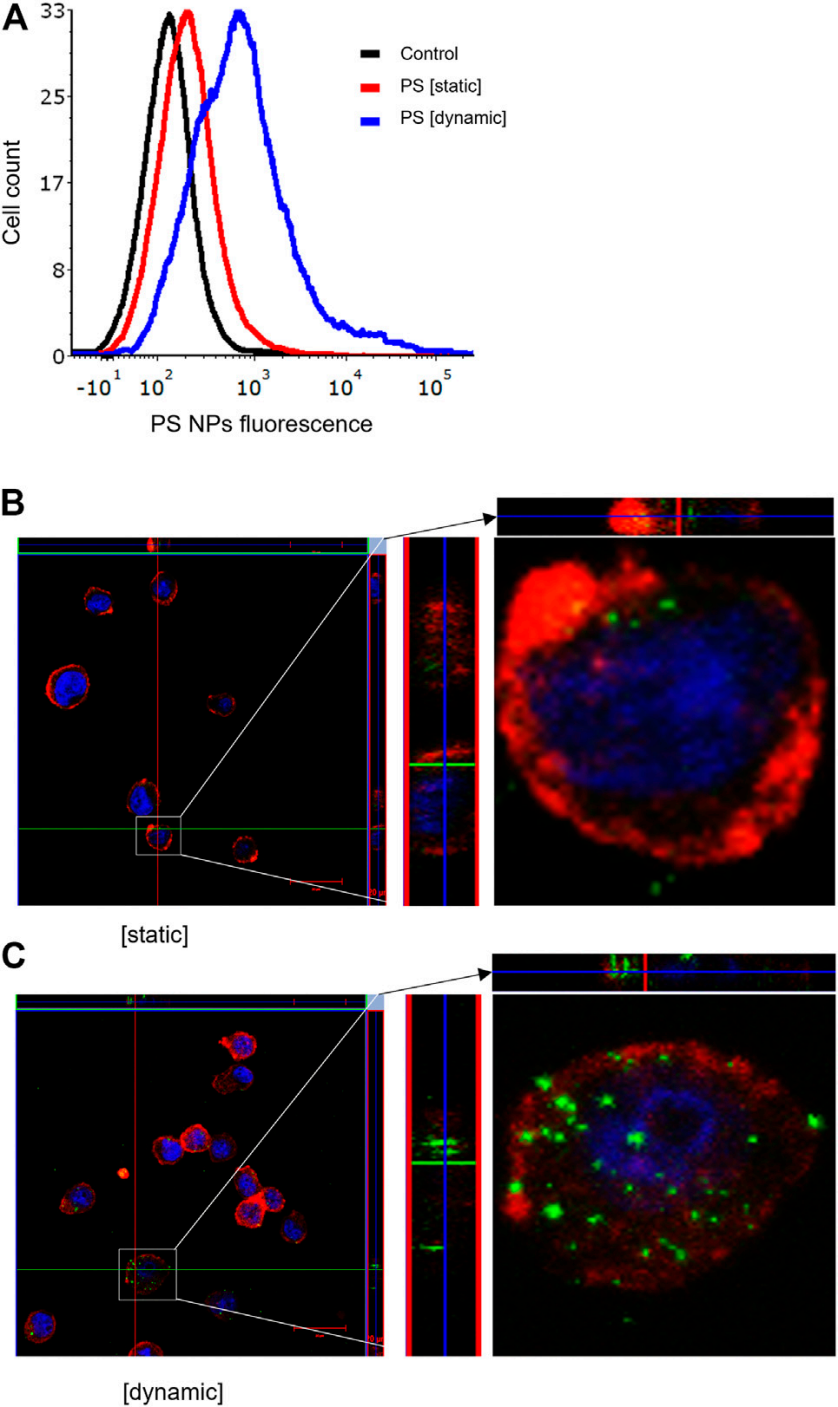
Cellular uptake of fluorescent PS NPs (10 μg/ml) under static and dynamic exposure conditions. **(A)** Cellular uptake of PS NPs determined using flow cytometry after 2 h of exposure of BEAS-2B cells **(B,C)** Confocal microscopy of cells exposed for PS NPs after 1 h under static and dynamic exposures, respectively. The z-stack imaging confirmed the intracellular presence of the NPs. Cells are counterstained using phalloidin red and DAPI (blue) to visualize the actin cytoskeleton and cell nucleus.

It is well established that size, shape, and surface properties of NPs are key determinants for NP interactions with cells and tissues. Furthermore, the density of the particles may also influence the likelihood of cellular interactions, as discussed above. We asked whether human bronchial epithelial cells would take up NPs of varying densities to a different extent under static vs. dynamic exposure conditions. To this end, SiO_2_ NPs and PS NPs were used as model NPs. The SiO_2_ NPs have a density roughly twice that of blood while the PS NPs are neutrally buoyant (Thompson and Eniola-Adefeso, 2015). BEAS-2B cells were briefly exposed in a conventional, static cell culture model vs. the previously established microfluidic-based COC and uptake was determined using flow cytometry and confocal microscopy. As shown in Figure 3A, uptake of the SiO_2_ NPs was identical at 2 h under dynamic conditions when compared to static exposure, and this was confirmed by confocal microscopy which revealed ample internalization of clusters of fluorescent NPs under both conditions (Figures 3B,C). In the case of the PS NPs (displaying a similar surface charge, but a lower density when compared to the SiO_2_ NPs), we observed limited uptake at 2 h under static conditions whereas particle uptake was enhanced under dynamic conditions (Figure 4A). This was confirmed by confocal microscopy (Figures 4B,C). Cellular internalization of the PS NPs was barely seen under static conditions (at 1 h) (Figure 4B). Thus, dynamic exposure to PS NPs enhanced the uptake of the otherwise buoyant NPs.

**FIGURE 5:**
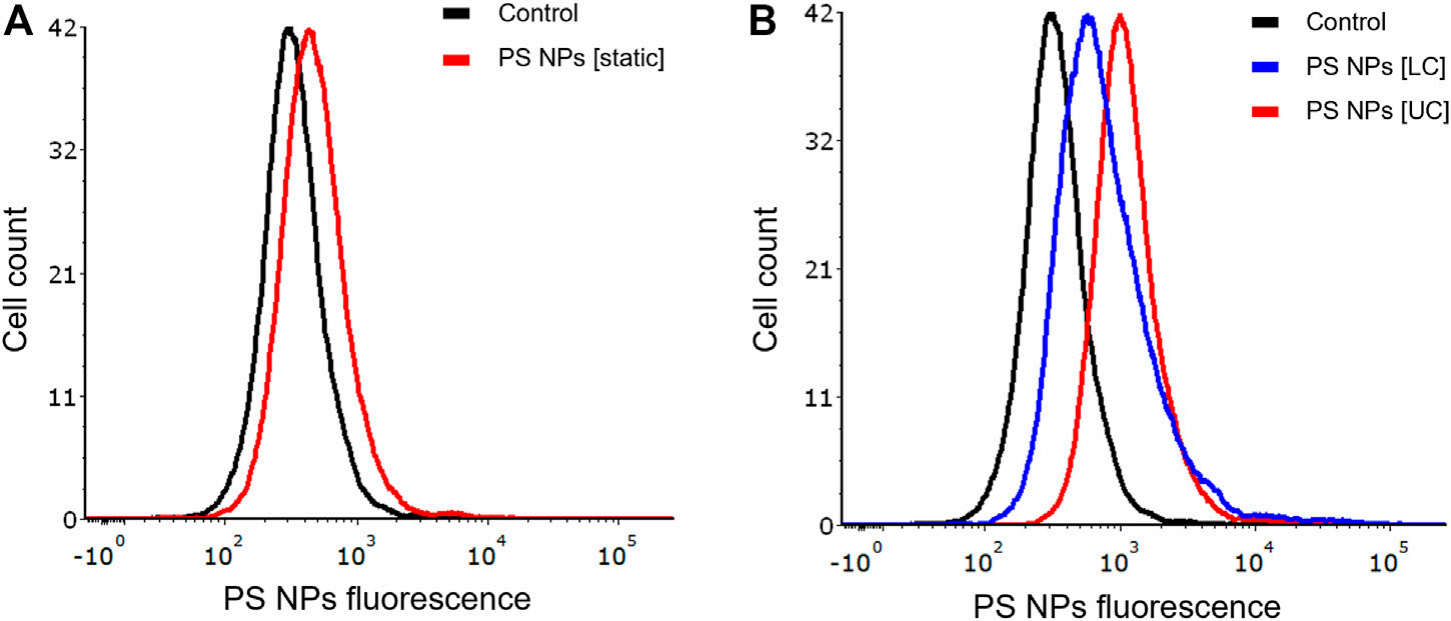
Role of cell polarity. BEAS-2B cells showed differential uptake of PS NPs (10 μg/ml) upon exposure through the upper and lower channels of the microfluidic device. **(A)** static conditions (conventional 24-well plate); **(B)** dynamic exposure using the COC platform. Cells were exposed for 1 h and the samples were analyzed by flow cytometry.

It is common knowledge that epithelial cells display polarity, characterized by apical and basolateral membrane domains separated by cell junctions. These adherens and tight junctions connect neighboring epithelial cells, while the basal surface interacts with the extracellular matrix through integrin receptors. The apical and basolateral membranes differ in terms of their protein and lipid composition (Cao et al., 2012). We investigated whether apical or basal exposure of NPs under dynamic conditions would influence cellular uptake of NPs. To this end, we exposed BEAS-2B cells in the COC device separately through the upper or lower flow chambers vs. under static conditions. We confirmed that there was less uptake of the PS NPs under static conditions (Figure 5A). Furthermore, we observed higher uptake of NPs in cells exposed *via* the upper chamber when compared to the lower chamber of the COC device (Figure 5B).

## Discussion

Most cell culture models are static, and do not reflect the dynamic conditions in a living system. The buoyancy of particles with densities lower or equal to that of cell culture medium poses a problem as they may not reach the cells at the bottom of the dish, leading to an underestimation of their effects on cells (Watson et al., 2016; Stock et al., 2020). Furthermore, several recent studies have shown that the cellular interactions of NPs are dictated by flow rate (Moore, et al., 2017; Yazdimamaghani et al., 2018; Chen et al., 2020). The size and shape of the particles may also come into play (Geng et al., 2007). However, the preferential interaction with spherical vs. elongated particles may be cell type-dependent, as demonstrated in an elegant recent study (Safari et al., 2020). In the present study, we focused on the density of the particles while shape and diameter were comparable. Hence, we tested amorphous SiO_2_ NPs (45 nm) and polystyrene (PS) NPs (50 nm), both displaying a negative surface charge in cell culture medium. However, it is important to note that the two tested NPs also may differ in other ways for instance with respect to their surface properties, which could affect the subsequent interactions of NPs with cellular receptors. The detection of nano- and microplastics in biotic and abiotic matrices remains a challenge (Mariano et al., 2021). In the present study, we applied fluorescently labeled NPs for the evaluation of cellular uptake by flow cytometry and confocal microscopy. The two different NPs were internally labeled meaning that the surface properties were not affected by the fluorophore while leaching of the fluorophore is prevented. We used the immortalized human bronchial epithelial cell line BEAS-2B as a model, and the cells were cultivated in serum-free medium; serum, after all, is not a natural biological element of the airways. Overall, our findings suggest that the mode of exposure (i.e., static vs. dynamic) should be considered in order to draw conclusions concerning low-density NPs.

As discussed above, Watson et al. (2016) found that nano-sized polypropylene (PP) particles were cytotoxic only when using an inverted cell culture platform. Similarly, Stock et al. (2020) developed an inverted *in vitro* cell culture system to test micrometer-sized polyethylene (PE) particles and demonstrated that the particles became cytotoxic to HepG2 cells only when exposed in “overhead” cell cultures. Here, we could show higher cellular uptake of PS NPs under dynamic exposure conditions while cellular uptake of SiO_2_ NPs was similar under static and dynamic conditions. The present study addressed the cellular uptake of NPs, and the different NPs were tested at a relatively low dose (10 μg/ml) up to 2 h of exposure to avoid overt cell death. However, it is relevant to ask whether enhanced cellular uptake of so-called nanoplastics under dynamic exposure conditions also translates into a cytotoxic response. Using a microfluidic device, Oddo et al. (2021) found that the exposure of a human B cell leukemia cell line to polyvinylpyrrolidone (PVP)-coated silver NPs under dynamic conditions resulted in a 3-fold increase in toxicity compared to static conditions. Further studies are needed to address whether this also holds true for so-called nanoplastics.

We have previously shown, using primary human monocyte-derived macrophages as a model, that surface coating (i.e., the intrinsic “identity”) as well as protein adsorption (the acquired “identity”) both affect the cellular uptake of magnetic NPs (Vogt et al., 2015). Furthermore, other investigators have shown that protein corona formation on the surface of lipid NPs is influenced by dynamic flow conditions, which may, in turn, affect uptake of the NPs in various cancer cell lines (Palchetti et al., 2017). In a more recent study, Srivastava et al. (2020) established a microfluidic-based system for the real-time monitoring of protein corona formation using carbon NPs displaying different surface properties. However, we maintained the cells in FBS- and BPE-free cell culture medium. This does not preclude the formation of a bio-corona derived from the cellular secretome, although this remains purely hypothetical at present. In fact, our DLS measurements at the inlet vs. the outlet of the microfluidic device demonstrated that the hydrodynamic diameter of the PS NPs was identical before and after exposure. Therefore, we may conclude that the differences in uptake of PS NPs under static and dynamic conditions is related to the flow and not to corona formation (Tenzer et al., 2013). It is pertinent to note that shear stress may also affect the cells themselves, and not only the way in which particles interact with the cells. Hence, Kim et al. (2011) investigated silica NPs using a microfluidics system and found that the NPs showed higher toxicity towards endothelial cells under flow conditions. The authors argued that these differences resulted from the shear stress rather than dose, one potential explanation being that increased shear stress triggers the activation of endothelial cells.

Bronchial epithelial cells serve as a barrier to pathogens and these cells are endowed with innate immune receptors including the so-called Toll-like receptors (TLRs) (Gliga et al., 2020). It is currently not known how nanoplastics gain access to cells, and whether specific cell surface receptor(s) are involved. However, we provided evidence suggestive of selective uptake of PS NPs *via* the apical cell membrane of BEAS-2B cells as opposed to the basolateral surface. This is not surprising as the apical membrane is normally confronted with the external environment (Cao et al., 2012). Further studies are needed to explore differences in endocytosis or phagocytosis of NPs at the apical and basolateral membranes in bronchial epithelial cells. It also remains to be proven whether BEAS-2B cells are truly polarized (Papazian et al., 2016). Previous studies have shown that uptake of simian virus 40 (SV40) by polarized epithelial cells is restricted to the apical membrane implying that its receptors are non-uniformly expressed in polarized cells (Clayson and Compans, 1988; Basak et al., 1992). Furthermore, several TLRs are expressed in epithelial cells of the intestinal tract. Interestingly, Lee et al. (2006) found that activation of TLR9 through the apical and basolateral surface of intestinal epithelial cells leads to distinct responses. This illustrates the critical importance of polarity of epithelial cells and shows that receptors may be expressed differently at the apical and basolateral membranes or, alternatively, that the same receptor may trigger distinct responses depending on the polarity of the cells.

## Conclusions

It has been shown that plastic particles float in cell culture medium and thus do not reach the cells under standard *in vitro* exposure conditions. We established a microfluidic-based platform that allows for dynamic exposure of cells. The human lung cells were maintained under completely animal-free conditions (*i.e.*, no animal-derived products such as fetal bovine serum or antibodies were used). Using this model, we studied the uptake of SiO_2_ NPs and PS NPs in cells under dynamic and static exposure. We observed higher uptake of PS NPs under dynamic conditions. These findings suggest that exposure conditions need to be adjusted to mimic the physiological conditions of shear stress especially when dealing with low-density particles. This is relevant not only for the safety assessment of nano- and microplastics, but also in nanomedicine, as shear stress may also dictate the interaction of drug-loaded NPs with cancer cells (Tee et al., 2019). Thus, standard *in vitro* methods based on static cell culture may not be suitable for studies of low-density (buoyant) particles, and may, in fact, underestimate the cellular uptake/impact of such particles, as shown here.

## Data Availability

The raw data supporting the conclusions of this article will be made available by the authors, without undue reservation.
