# Supplementary material for: Development of Microfluidic, Serum-Free Bronchial Epithelial Cells-on-a-Chip to Facilitate a More Realistic In vitro Testing of Nanoplastics
Source: Front Toxicol. 2021 Oct 6;3:735331. doi: 10.3389/ftox.2021.735331 (PMC8915849; doi:10.3389/ftox.2021.735331)
Supplement: Supplementary file 1 [file DataSheet1.PDF]

Supporting Information

Development of microfluidic, serum-free bronchial epithelial cells-on-a-chip to facilitate a more realistic *in vitro* testing of nanoplastics

Govind Gupta[1], Srikanth Vallabani[2], Romain Bordes[3], Kunal Bhattacharya[1], and Bengt Fadeel[1]

[1]Unit of Molecular Toxicology, and [2]Unit of Biochemical Toxicology, Institute of Environmental Medicine, Karolinska Institutet, Stockholm, Sweden; [3]Department of Chemistry and Chemical Engineering, Chalmers University of Technology, Göteborg, Sweden.

Table S1: BEAS-2B cells grown in 24-well culture plates *versus* in a microfluidic chip.

culture vessel	growth area	seeding density[a]	cell death[b]
24-well plates	1.9 cm^2	1.2 x 10^5 cells/well	5.6 ± 0.4
microfluidic chip	1.6 cm^2	1.0 x 10^5 cells/well	6.7 ± 4.9

[a]BEAS-2B cells were seeded at 60.000 cells/cm^2 in 24-well plates or microfludic chips.

[b]Cell death was determined 24 h after seeding using the LDH release assay (% LDH release compared to max. lysis of the cells) . Data shown are mean values ± S.D.

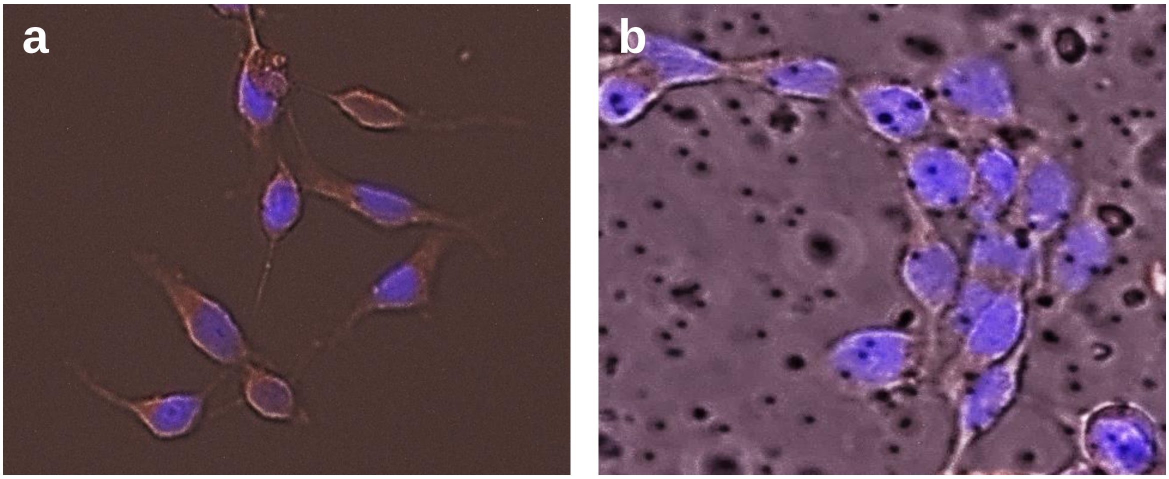

Figure S1: Morphology of the BEAS-2B cells grown in a 24-well culture plate (A) *versus* a microfluidic chip (B). The PET membrane pores are seen in (B). Images were captured at 400x magnification 24 h after seeding of the cells. Nuclei were visualized using DAPI (blue) and the plasma membrane is demarcated with CellMask™ Deep Red.

Box 1: Microfluidics setup for the dynamic exposure of nanoparticles or nanoplastics in lung cells.

The system comprises of pressure controller, pressure source, inlet/outlet reservoirs, microfluidics unit, and lung cell cultures-on a-chip (COC). For details on the COC, refer to Box 2.

Pressure controller:

- Positive pressure is connected *via* the indicated ports, passing towards the humidity filter to the flow controller. Note that there are three individual ports. Port 1 is used to attain an output pressure of >1.0 bar to 10 bar. Port 2 and 3 are used for setting 1 bar output pressure, respectively.

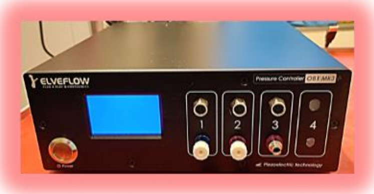

Microfluidic flow controller

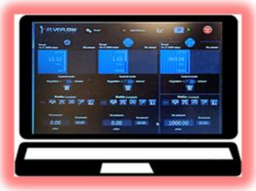

Elveflow smart interface

Caution:
1) Close the pressure port with a luer lock when not in use. The maximum applied pressure should not exceed 10 bar for port 1 and 1 bar for port 2 and 3.
2) A 0.22 µm filter can be interconnected between pressure source and flow contoller to ensure air sterility.

- A positive pressure is applied in the form of clean air, which passes through the humidity filter.

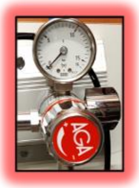

Pressure source

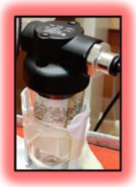

Particle/Humidity filter

Caution:

Filter must be placed between the pressure source and controller in order to avoid damage to the instrument from incoming particles or moisture.

- The pressure controller is operated through a dedicated software (Elvesys), which maintains a known pressure between 1-10,000 mbar to achieve the desired flow rate through the chamber.

Microfluidics unit:

- Inlet and outlet are connected to the microfluidic unit to inject and collect samples from the COC (Micronit).
- Two separate connections are established to maintain flow in both the upper and lower compartments.

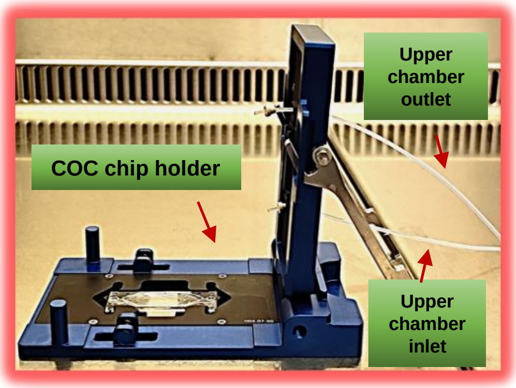

Microfluidics unit

Critical steps:

1) Carefully connect the inlet and outlet tubing, to avoid mixing of the samples collected from the upper and lower chambers.

2) Gently lock the chip holder while sealing the COC chips to avoid breaking the COC layers (note: especially the lower layer).

Box 2: Cell culture-on-a-chip setup for the analysis of nanoparticles/plastics under flow conditions.

Cell culture-on-a-chip:

- BEAS-2B cells are seeded on the membrane cavity of the middle cell culture layer and allowed to adhere for 24 h. The cells are maintained in serum-free culture medium (see main text).

> **Note:** The setup allows for the cellular contact with NPs from both apical and basolateral cell membranes, unlike conventional cell culture conditions (i.e., cells in a culture dish).

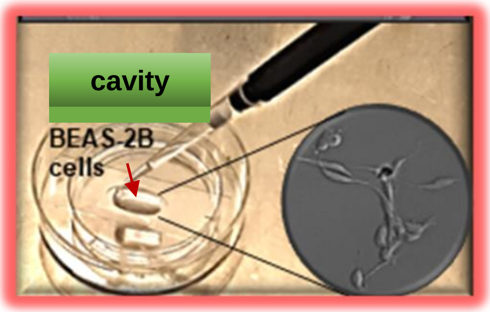

Bronchial epithelial cells

Assembling the chip:

- To create upper and lower chambers, the middle cell culture layer is sandwiched between the two.

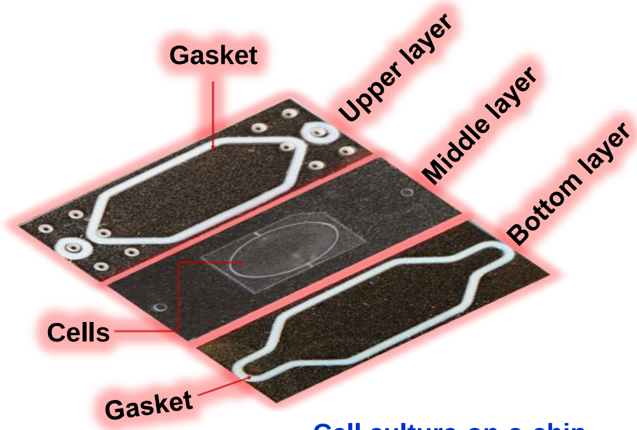

Cell culture-on-a-chip

> **Critical steps:**
>
> 1) Observe the cells in the middle culture layer under the microscope before sealing.
> 2) Gaskets in the upper and lower layer should face towards the middle cell culture layer, to ensure tight sealing and the formation of the upper and lower chambers.

Cell processing:

- After exposure, the COC layers are gently separated, removing the extra medium from the cavity, and cells are trypsinized to detach from the membrane. Note that cells should be observed under the microscope after trypsinization to ensure that all the cells have been collected.
- Cells are then fixed in 4% paraformaldehyde and/or processed for further analysis as required.

Endpoint analysis:

- Nanoparticle uptake by cells is determined by flow cytometry.

Furthermore, confocal microscopy can be done to prove cellular internalization of the particles (cell nuclei counterstained with DAPI).

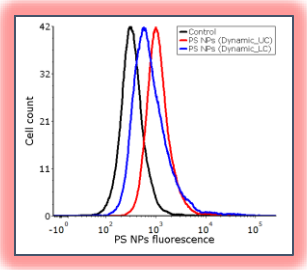

Flow cytometric analysis

Disclaimer: The authors have no financial or other relation or agreement with Elvesys or Micronit.
